# Collagenase treatment reduces the anisotropy of ultrasonic backscatter in rat myocardium by reducing collagen crosslinks

**DOI:** 10.14814/phy2.15849

**Published:** 2023-11-13

**Authors:** Lindsay A. Pittman, Peter Whittaker, Michelle L. Milne, Charles S. Chung

**Affiliations:** ^1^ Department of Physiology Wayne State University Detroit Michigan USA; ^2^ Green Templeton College University of Oxford Oxford UK; ^3^ Department of Physics St Mary's College of Maryland St Mary's City Maryland USA

**Keywords:** anisotropy, echocardiography, fibrosis, imaging, picrosirius red

## Abstract

Dysregulation of collagen deposition, degradation, and crosslinking in the heart occur in response to increased physiological stress. Collagen content has been associated with ultrasonic backscatter (brightness), and we have shown that the anisotropy of backscatter can be used to measure myofiber alignment, that is, variation in the brightness of a left ventricular short‐axis ultrasound. This study investigated collagen's role in anisotropy of ultrasonic backscatter; female Sprague–Dawley rat hearts were treated with a collagenase‐containing solution, for either 10 or 30 min, or control solution for 30 min. Serial ultrasound images were acquired at 2.5‐min intervals throughout collagenase treatment. Ultrasonic backscatter was assessed from anterior and posterior walls, where collagen fibrils are predominately aligned perpendicular to the angle of insonification, and the lateral and septal walls, where collagen is predominately aligned parallel to the angle of insonification. Collagenase digestion reduced backscatter anisotropy within the myocardium. Collagen remains present in the myocardium throughout collagenase treatment, but crosslinking is altered within 10 min. These data suggest that crosslinking of collagen modulates the anisotropy of ultrasonic backscatter. An Anisotropy Index, derived from differences in backscatter from parallel and perpendicularly aligned fibers, may provide a noninvasive index to monitor the progression and state of myocardial fibrosis.

## 1 INTRODUCTION

The need for earlier detection of myocardial fibrosis, as well as the science suggesting that ultrasound may hold the capability to do so, is well‐established (Hassan et al., 2020). However, the physiological link between ultrasound and collagen structure remains missing (Buckberg et al., 2004). This study aims to bridge that gap, as well as propose a potentially widely applicable quantitative index of anisotropy.

Clinically, echocardiographic indexes derived from Doppler flow and tissue imaging or strain rate are used to assess myocardial fibrosis (Baues et al., 2017). In recent studies, myocardial backscatter has been proposed as a method to characterize fibrotic deposition (Baues et al., 2017; de Jong et al., 2011; O'Donnell et al., 1981). These studies typically assess backscatter (brightness) of only the posterior myocardium from the parasternal long‐axis view (Aygen & Popp, 1987; Papadacci et al., 2017). In short, the intensity of ultrasonic backscatter in cardiac muscle (and other striated muscle) shows anisotropy (variation) subject to the angle in which it is viewed, and is associated with myofiber orientation (Aygen & Popp, 1987; Holland et al., 2005; Khan et al., 2008). The highest backscatter intensity is generated where myofibers are aligned perpendicular to the angle of insonification and is lowest where myofibers are parallel. We, and others, have shown that anisotropy of ultrasonic backscatter relates directly to the alignment of myofibers, evidenced in both short and long‐axis ultrasonic images, of small and large mammals (Holland et al., 2005; Milne et al., 2012, 2016, 2019). Thus, myocardial backscatter is anisotropic, or variable, depending on the orientation of the ultrasound source and the myocardial myofiber (mesostructure) alignment. To date, there is no known intracellular or extracellular component that explains this anisotropy.

We hypothesize that extracellular properties of myofibers, particularly the integrity of the muscles' collagen network, dominates backscatter anisotropy in the myocardium and begets the ability to be quantified. To investigate the relationship between anisotropy of ultrasonic backscatter and properties of collagen, we utilized collagenase digestion protocols commonly used for myocyte isolation (Czeiszperger et al., 2020; Louch et al., 2011). The serial ultrasound images taken during the digestion process provide a means to quantify the relationship between collagen properties and the anisotropy of the myocardium.

## 2 METHODS

### 2.1 Ex vivo ultrasonic imaging

A total of 22 female Sprague–Dawley (Charles River, Wilmington, MA) rats aged 16.7 ± 2.3 weeks were used in this study. Sex was not considered a biological variable because a treatment (collagenase) effect on a biological sample (heart) was being assessed; use of more than one sex could have led to unnecessary duplication if the treatment in this initial study did not produce an effect. The study utilized female rates for consistency with previous studies by our lab group when assessing backscatter intensity anisotropy (Milne et al., 2019). Animal use was approved by the Institutional Animal Use and Care Committee of Wayne State University. Animals were anesthetized by inhalation of 3%–4% isoflurane and euthanized by exsanguination. Hearts were quickly excised and rinsed in cold (4°C), oxygenated, HEPES‐buffered Perfusion Solution (PS) (in mmol/L: 113 NaCl, 4.7 KCl, 0.6 KH2PO4, 1.2 MgSO4, 12 NaHCO3, 10 KHCO3,10 4‐(2‐hydroxyethyl)‐1‐piperazineethanesulfonic acid [HEPES], 30 Taurine, 5.5 glucose, and 10 2,3‐butanedione monoxime [BDM]; pH ~7.3). Hearts were transferred to a custom 3D printed experimental chamber filled with isotonic solutions (PS and phosphate buffered saline [PBS]). Isolated hearts were cannulated via the aorta and attached to a gravity fed Langendorff perfusion system and perfused with 37°C, continuously oxygenated PS to clear blood from the coronary vessels. Flow reservoirs were stationed at approximately 70 cm above the experimental chamber to ensure coronary perfusion.

A VisualSonics MS250D transducer (21‐Mhz center frequency) attached to a Vevo3D motor (FujiFilm VisualSonics, Toronto, ON) was positioned transversely over the mid‐ventricle of isolated hearts, allowing for a parasternal short axis view. The transducer was connected to a VisualSonics Vevo 2100 small animal ultrasound system. Receiver gain was set to 60 dB and time gain compensation (TGC) was set to zero for each heart. These gain settings were previously established to maintain a linear relationship between backscatter and gain (Milne et al., 2019). Once the transducer was aligned to obtain short axis (parasternal‐like alignment) 3D images, the experimental procedure began. Control hearts (CTRL, *n* = 8) were continuously perfused with PS for 30 min. For collagenase treated hearts, PS was immediately changed to a collagenase solution (PS containing 2 μmol/L CaCl_2_ and 235 U/mL Collagenase Type II, (CSL‐2 Collagenase, Worthington Biochemical, Lakewood, NJ)) for either 10 or 30 min (*n* = 7 for each treatment). The change from PS to collagenase solution was used as timepoint 0. Solutions in flow reservoirs were continuously oxygenated. Serial, 3D, B‐mode images were acquired at 2.5‐min intervals after obtaining the initial, aligned image.

After treatment with PS with collagenase solution or PS, hearts were separated and prepared for histology and biochemistry. Briefly, atria and vasculature were discarded with a transverse cut directly below the atria, and a transverse section of ventricular tissue approximately 2 mm thick near the middle of the heart was flash frozen in liquid nitrogen and stored at −80°C until used for biochemistry. A section of the apical tissue was placed in 4% paraformaldehyde and reserved for histology. One sample was excluded from biochemistry and one sample was excluded from histology as a result of error in tissue preparation. Due to the addition of BDM to our PS, it can be assumed that all hearts remained in a passive, isotonic state throughout the procedure, and thus were fixed or frozen in a passive state, as well.

### 2.2 Analysis of ultrasonic backscatter

Analysis was performed on a defined single short‐axis frame of the 3D data set for each heart at or near the middle of the heart, from apex to base; image slices that contained substantial chamber reflections or bubbles that could potentially influence accurate image analysis were avoided. VisualSonics RAW data for this slice at each timepoint (images taken 2.5 min apart) was exported using the VisualSonics software and analyzed using custom MATLAB scripts. A myocardial section half‐way between the endocardial and epicardial walls was analyzed at the lateral LV, septal, anterior LV and inferior LV walls. Anterior and inferior LV walls were assumed to contain fibers predominately perpendicular to the angle of insonification, while the lateral and septal walls were assumed to contain fibers predominately parallel to the angle of insonification. The mid‐myocardium of the left ventricle (LV) was located by manually mapping an ellipse to both the endocardial and epicardial walls at each timepoint. Backscatter intensity was quantified using a 250 × 250 μm^2^ area centered at these points. The wall thickness of the myocardium was calculated from the positions of the endocardial and epicardial shapes at all time points and averaged.

An Anisotropy Index was calculated by determining the absolute difference between locations assumed to be aligned perpendicular and parallel to the angle of insonification. Four measures of the Anisotropy Index were calculated from each image (anterior‐lateral, anterior‐septal, posterior‐lateral, posterior‐septal) and an average Anisotropy Index value was calculated.

### 2.3 Histological analysis by picrosirius red staining

Ventricular tissue designated for collagen staining was stored in 4% paraformaldehyde for at least 24 h at 4°C and then transferred to 30% sucrose in PBS solution until the tissue was fully infiltrated. Tissue was then embedded and stored in Optimal Cutting Temperature compound (OCT compound) at −20°C by the experimenter. Embedded tissue was sectioned at ~5 μm, mounted on to Fisherbrand Superfrost Plus slides, and stained with Picrosirius Red (iHi PSR‐IFU, iHisto, Salem, MA), blinded, by an external provider (iHisto, Salem, MA). After staining for 10 min, slides were dehydrated using an alcohol gradient, cleared with xylene, and sealed with a coverslip.

Picrosirius red‐stained slides were imaged on an Olympus BX51 microscope using a digital camera (DP11, Olympus) captured and analyzed using SigmaScan Pro image analysis software (SPSS Inc). The microscope has perpendicularly aligned linear polarizing filters above and below a rotating stage. For some of the analysis, circularly polarized light was used; produced by placement of a circularly polarizing filter below the microscope stage and the combination of a quarter wave‐plate and a linear polarizing filter above the stage. Image acquisition and analysis were performed by an experimenter blinded to the treatment condition.

To determine if the collagenase perfusion altered collagen content, slides were assessed using an image subtraction approach adapted from previously established methods (Lillian Rich, 2005; Whittaker et al., 1994). Briefly, tissues were imaged using a 40× objective lens and circularly polarized light. The background and non‐collagen tissue elements were removed by first resolving the original image in red, blue, and green components with an automated function provided by the software. The blue component image was subtracted from the original; this reduced the brightness of the muscle. Then, the original image was resolved into its cyan, yellow, magenta, and black components. Of these, only the black component was assessed and its value was subtracted from the first subtracted image. Collagen fiber hue in picrosirius red‐stained section viewed with polarized light depends upon fiber thickness (Hiss et al., 1988; Junqueira et al., 1982); the color changes from red to orange to yellow to green as thickness decreases. The image analysis software was used to provide the distribution of pixels with different hues in an 8‐bit image containing 256 colors. We used the following hue definitions; red/orange 2–38, yellow 39–51, and green 52–128. Collagen content was determined by summation of all the pixels in the range 2–128 divided by the total number of pixels in the image and expressed as a percentage. The proportion of each fiber color was calculated as a percentage of the total collagen content.

To determine if the collagenase treatment altered subcellular muscle molecular anisotropy, we measured muscle birefringence. Materials with an anisotropic structure are said to be birefringent; that is, they possess two refractive indexes and the greater the anisotropy, the greater the birefringence. The degree to which linearly polarized light is retarded by passing through a birefringent material depends upon the thickness of the material in addition to its birefringence (Wolman, 1970). Thus, if thickness is constant, retardation provides information on the material's anisotropy. For example, thermal injury results in loss of cardiac muscle anisotropy (Whittaker et al., 2000). We measured the retardation of muscle in each sample using the method of de Sénarmont (Whittaker et al., 2003). Ten retardation measurements were made in each section from longitudinally sectioned myocytes and the group averages (in nanometers) calculated.

To determine the architecture of treated myocytes, the optical properties of birefringent materials when viewed with linearly polarized light were exploited. In this way, we measure the myocytes' two‐dimensional orientation (Batschelet, 1981; Whittaker et al., 1989, 2003; Whittaker & Canham, 1991). This method has previously been used to quantify myocyte disarray in hypertrophic cardiomyopathy (Whittaker et al., 1989). One region that contained longitudinally sectioned muscle was analyzed in each sample. Fifty orientation measurements were made per sample in a 10 × 5 rectangular grid pattern using a 20× objective lens. For each sample, we calculated the angular deviation of the orientation distribution (Batschelet, 1981); the circular statistics equivalent of standard deviation. The average angular deviations of each group were compared. In addition, we combined all the data to construct orientation distributions for each group.

### 2.4 Collagen crosslinking by hydroxyproline

Collagen crosslinking was quantified using a commercially available colorimetric hydroxyproline assay kit (MAK008‐1KT, Sigma‐Aldrich, St Louis MO). In brief, quantification of collagen crosslinking for each sample was determined by colorimetric crosslinking assay, adapted from a previously described protocol (Haynes et al., 2014). To ensure uniformity between tissue samples, mid‐lateral LV tissue was cut from partially thawed samples and again flash frozen using liquid nitrogen. Approximately 20 mg of tissue was homogenized with deionized water, pipetted into ampules, and was hydrolyzed with equal parts HCl. Samples were hydrolyzed for 24 h at 120°C in sealed glass ampules, centrifuged, mixed with reagents, and loaded onto a 96‐well plate (MidSci, St. Louis, MO) according to vendor instructions. Absorbance was obtained at 560 nm using a spectrophotometer (SpectraMAX Plus and SOFTmax Pro, Molecular Devices, San Jose CA). Colorimetric data was reported as an average of the three wells for each sample and content was calculated in reference to the serially diluted standard.

### 2.5 Statistics

Statistical testing was performed using SPSS (version 28, IBM). Data were analyzed using a one‐way ANOVA for comparisons against treatment conditions or Generalized Linear Mixed Models for comparisons against treatment condition and hue or against treatment condition, myocardial position, and timepoint. All main effects and interactions were considered. The Mixed Model was equivalent to a multi‐factor ANOVA. Animal identifier and location in the heart were fixed subject variables, while timepoint was treated as a repeated measure. Multiple comparisons were performed using a sequential Bonferroni post hoc tests. An *α* = 0.05 was set a priori.

## 3 RESULTS

### 3.1 Collagenase treatment impacts ultrasonic backscatter

**FIGURE 1:**
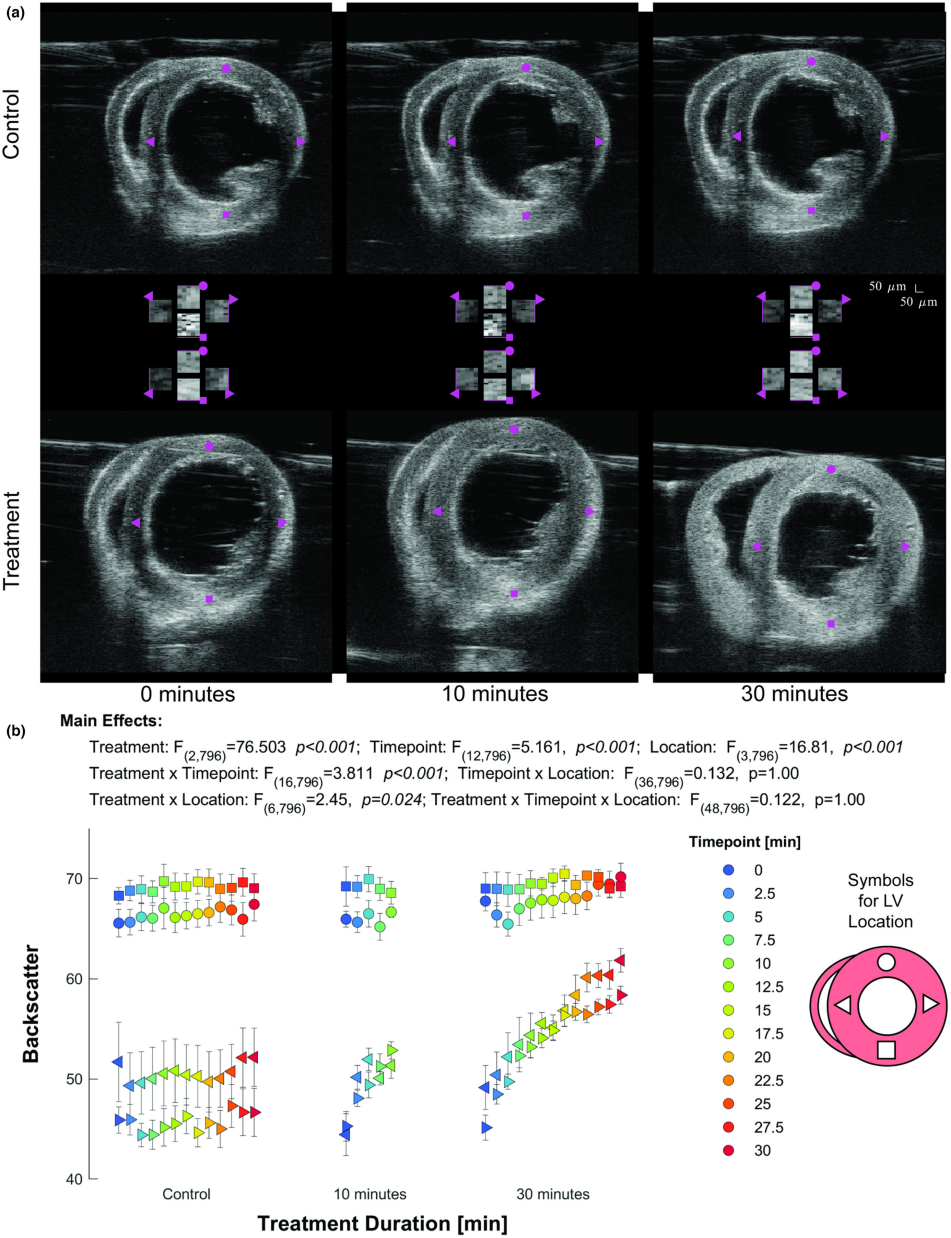
Effect of Collagenase Treatment on Ultrasonic Backscatter. (a) Mid‐myocardial short‐axis ultrasonic backscatter of one control (top) and one collagenase treated (bottom) heart at three time points. Horizontal center represents the generalized backscatter at each of the four cardinal positions analyzed. The LV positions as shown on the inset for (b) are: ●‐anterior myocardium; ►‐lateral myocardium; ■‐ posterior myocardium, ◄‐septal myocardium. Scale bar shows 50 μm square for full short‐axis views (top/bottom); center images are each 25 μm square. (b) Statistical analysis. Treatment duration and myocardial position show a significant interaction, with 30 min data suggesting an increase in backscatter intensity in myocardial sections that align parallel to the angle of insonification, and indicate minimal alteration in backscatter intensity in myocardial locations that are perpendicular to the direction of insonification. Treatment and treatment duration show a significant interaction, with controls showing little or no change in intensity over 30 min, while collagenase treatment increases backscatter intensity of septal and lateral wall positions (triangles) for each timepoint.

The impact of collagenase perfusion was visually apparent throughout the duration of the treatment (Figure 1). A three‐way Mixed Model was used to identify the relationships between the mean ultrasonic backscatter and treatment (collagenase duration), timepoint (time after treatment start), and location (myocardial position). The three‐way interaction was not significant (F_48,796_ = 0.757, *p* = 0.887). Two‐way tests revealed significant interactions between collagenase duration and myocardial position (F_6,796_ = 15.187, *p* < 0.001) and collagenase duration and timepoint (F_16,796_ = 2.183, *p* = 0.005). The two‐way interaction between timepoint and myocardial position was not significant (F_36,796_ = 0.991, *p* = 0.486).

Post hoc analysis revealed the specific differences in each two‐way interaction (see supplemental statistical output). The interaction between collagenase duration and myocardial position was evaluated using sequential Bonferroni corrections with position used as the contrast field. All data were statistically different, except the septal and lateral ventricular walls over the 10 min collagenase duration, which is consistent with anisotropy of ultrasonic backscatter seen in Figure 1. The two‐way interaction between collagenase duration and timepoint was evaluated using sequential Bonferroni corrections with the timepoint used as the contrast field. No differences at any timepoint were observed within either the control or 10 min collagenase duration groups. Within the 30 min collagenase duration group, backscatter magnitude at and following the 15 min timepoint differed from the starting backscatter value (0 min timepoint). The last (30 min) timepoint was not significantly different from the 10 min timepoint.

Wall thickness was quantified to ensure that the collagenase did not induce a change in the thickness or tissue density, which could be a confounding variable for the ultrasound backscatter. A three‐way Mixed Model was used to identify the relationship between wall thickness and treatment, timepoint, and location. No interaction was statistically significant but mean wall thickness was statistically different by treatment (F_2,199_ = 35.269, *p* < 0.001), but not timepoint (F_12,199_ = 0.632, *p* = 0.814), or their interaction (F_16,199_ = 0.181, *p* = 1.0). The lack of significance in timepoint and interaction indicates the collagenase treatment did not impact the wall thickness. The post hoc test for treatment suggests that hearts in the control group had thinner walls versus the collagenase groups (*p* < 0.001).

### 3.2 Anisotropy Index

**FIGURE 2:**
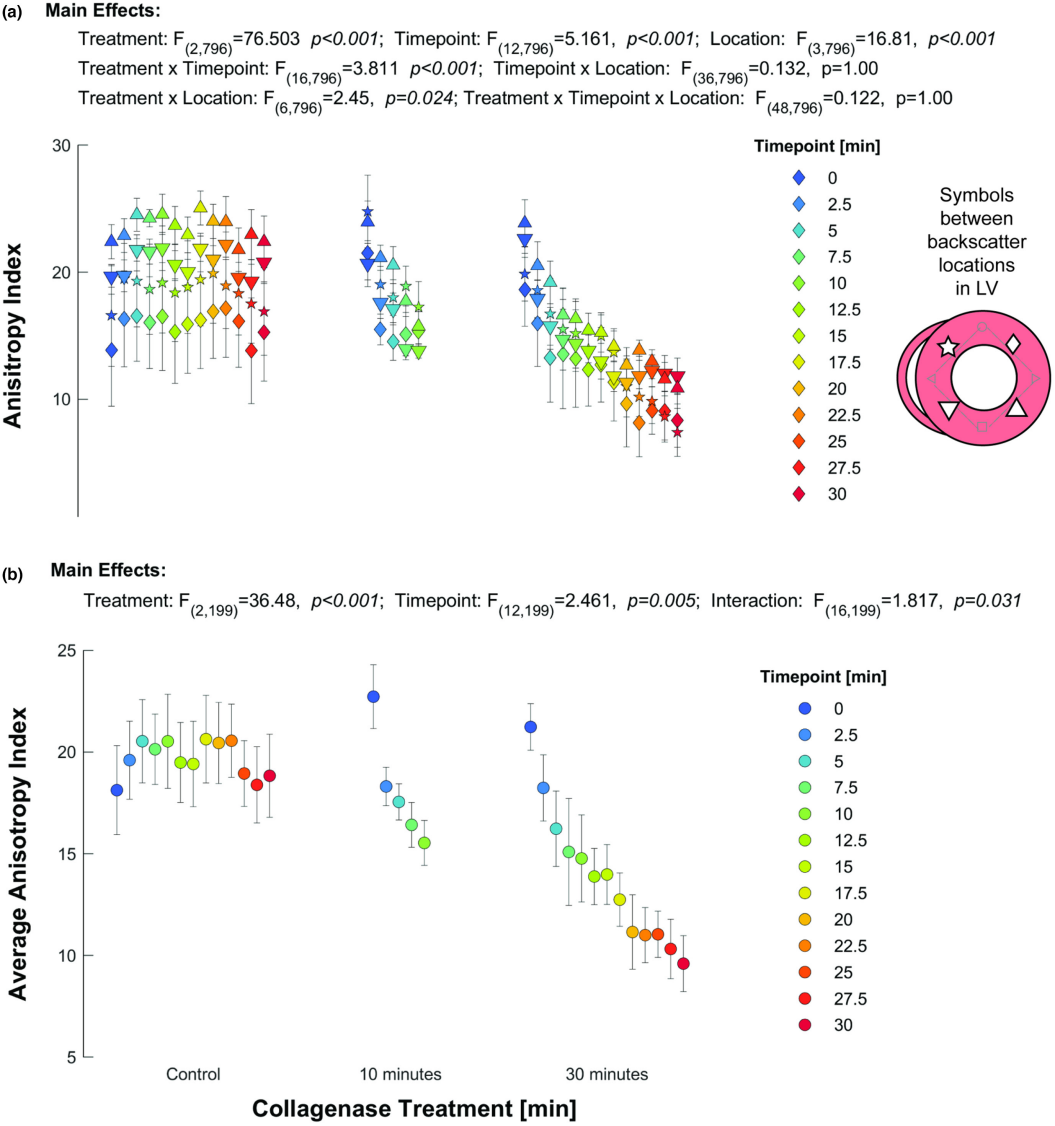
Anisotropy Index. (a) The anisotropy index was determined from four combinations from the difference in backscatter at the four cardinal positions (Figure 1b). Symbols, with gray lines connecting the originating data positions: ♦‐anterior minus lateral; ▲‐posterior minus lateral; ▼‐ posterior minus septal, ★‐ anterior minus septal. (b) The Average Anisotropy Index was calculated from the mean absolute difference of the anisotropy index, that is, approximately the magnitude of the gap between the anterior or posterior walls and the lateral and septal walls. The anisotropy index shows no change over time in the control but is reduced as the tissue is treated with collagenase solution.

Because total backscatter in myocardial tissue may be impacted by a number of factors, an Anisotropy Index was developed using differences from the raw backscatter data of each heart (Figure 2). A three‐way Mixed Model was used to identify the relationships between the Anisotropy Index and treatment, timepoint, and location. The three‐way interaction was not significant (F_48,796_ = 0.122, *p* = 1.0). Two‐way tests revealed significant interactions between collagenase duration and timepoint (F_16,796_ = 3.811, *p* < 0.001) and collagenase duration and myocardial position (F_6,796_ = 2.405, *p* < 0.024). The two‐way interaction between timepoint and myocardial position was not significant (F_36,796_ = 0.132, *p* = 1.0).

The two‐way interaction between collagenase duration and myocardial position was evaluated using sequential Bonferroni corrections with position used as the contrast field (see supplemental statistical output). For the control data, all individual Anisotropy Indexes were significantly different, suggesting some heterogeneity throughout the heart. For the 10 min collagenase duration, all Anisotropy Indexes were not statistically different, suggesting that the backscatter differences were becoming more homogenous. For the 30 min collagenase duration, only the posterior minus lateral wall comparison showed significant differences, suggesting septal and lateral wall treatment may differ over time. The two‐way interaction between collagenase and timepoint was evaluated using sequential Bonferroni corrections with timepoint used as the contrast field. Control data showed no significant difference in Anisotropy Index over time. For the 10 min collagenase duration, Anisotropy Index was altered within 5 min, while the 30 min collagenase duration was significantly altered by the 7.5 min timepoint. The average Anisotropy Index (Figure 2b) for each heart at each time point similarly suggests that the Anisotropy Index is dependent on treatment.

### 3.3 Histological analysis of collagen

**FIGURE 3:**
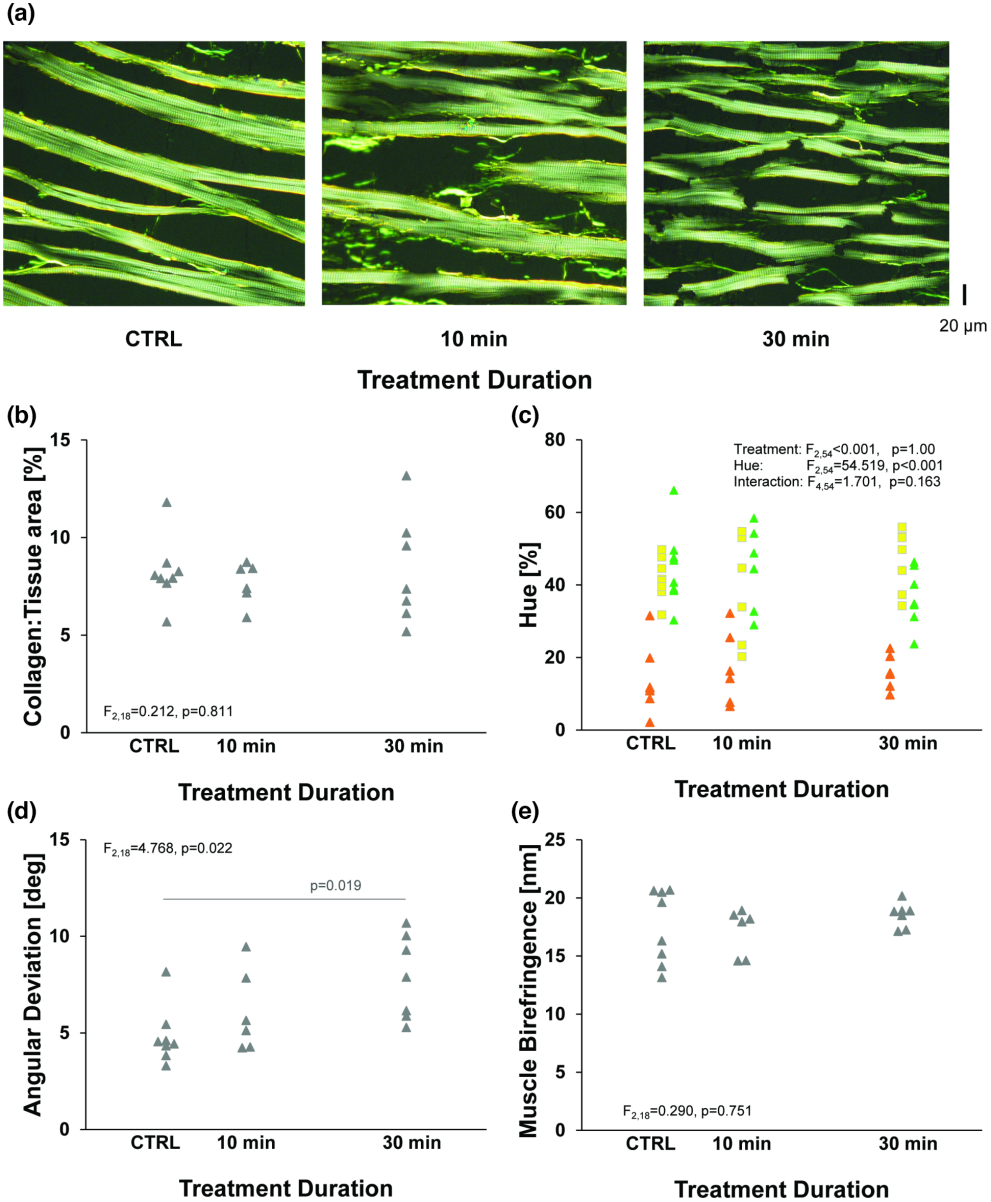
Histological Analysis of collagen. (a) Example circularly polarized light micrographs of hearts in the three treatment groups. Visible tinseling after 10 min of collagenase treatment. (b) Collagen to tissue ratio for the three treatment groups showing no change in collagen content, suggesting the amount of collagen is not changing over the course of the treatment. (c) Hue was used to estimate fiber size (orange thick, green thin). The relative percent of the hues differed, but hue was not dependent on treatment. (d) Angular deviation shows a dependence on treatment, suggesting that disarrangement of the collagen throughout the course of study treatment. (e) Myocyte (Muscle) Birefringence for the three treatment groups, showing no change, indicating no damage to myocytes.

Analysis of Picrosirius red‐stained sections indicated that collagen content was unchanged by the collagenase treatment, but muscle orientation did change (Figure 3). The collagen to tissue area ratio shows no significant change by treatment (one‐way ANOVA, F_2,18_ = 0.212, *p* = 0.811, Figure 3b). To investigate whether the hue of polarized light from collagen differed in each treatment, a two‐way linear mixed model was used. The treatment factor was not statistically significant (F_2,54_ = 0.000, *p* = 1.00), although the proportion of the three hues (green, yellow, red/orange) were different. As expected (F_2,54_ = 54.519, *p* < 0.001), the interaction did not reach the α threshold (F_4,54_ = 1.701, *p* = 0.163; Figure 3c). Collagenase includes several proteases, but myocyte (muscle) birefringence data also suggests there was no substantial myocyte degradation (one‐way ANOVA, F_2,18_ = 0.290, *p* = 0.751, Figure 3d). Qualitative visual inspection indicated that muscle cells appeared disorganized after collagenase treatment. The angular deviation of alignment indicates a treatment dependence (one‐way ANOVA, F_2,18_ = 4.768, *p* = 0.022, Figure 3e) with a post hoc test between the 0 and 30 min collagenase treatment groups being statistically significant (*p* = 0.019).

### 3.4 Collagen crosslinking

**FIGURE 4:**
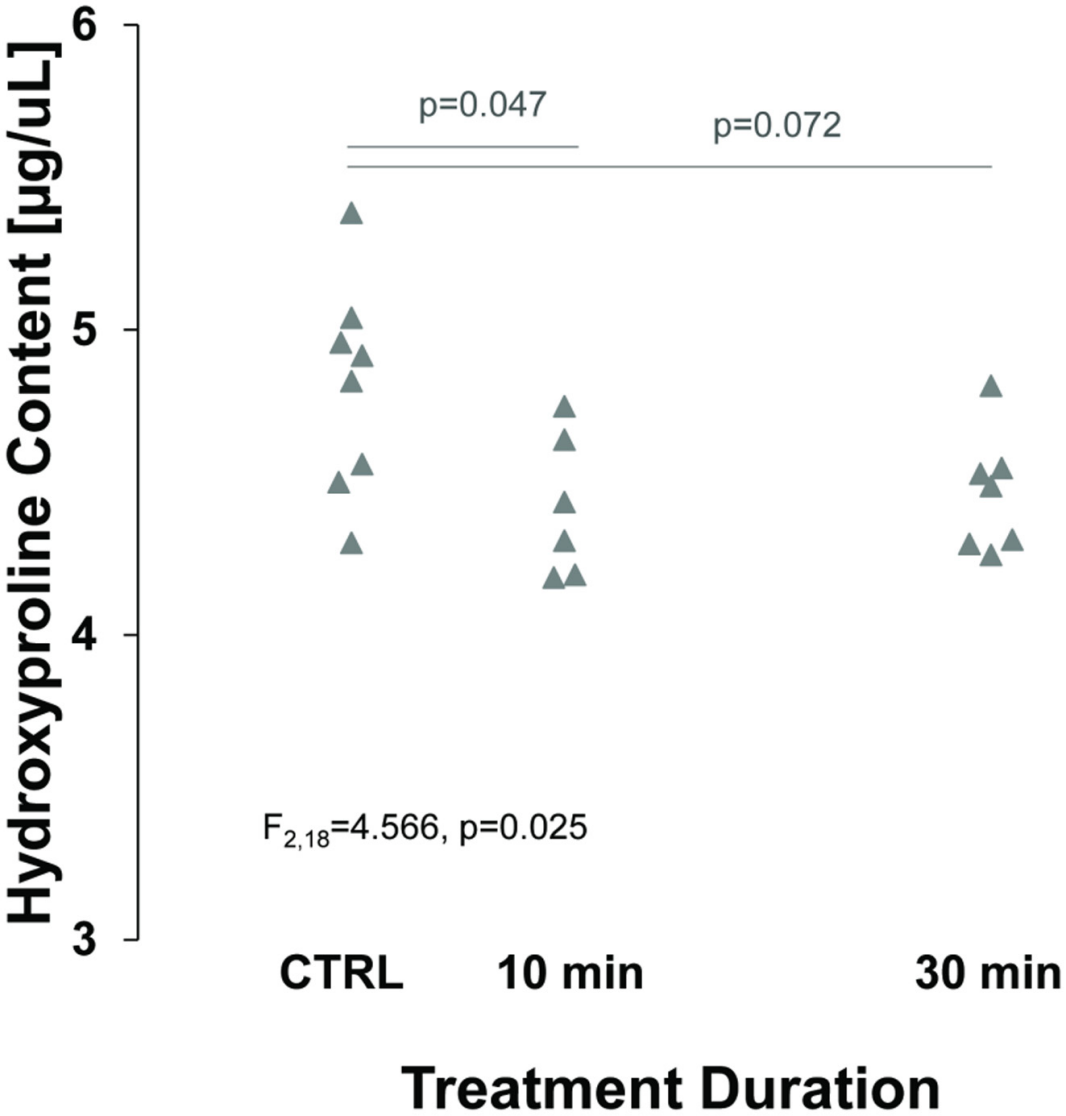
Hydroxyproline content in the three treatment conditions showing a reduction in crosslinking after collagenase treatment, significant after 10 min.

A colorimetric hydroxyproline assay was used to quantify collagen crosslinking in the three experimental conditions (CTRL *n* = 8, 10 min. *n* = 6, 30 min. *n* = 7). A one‐way ANOVA revealed a significant Main Effect (F_2,18_ = 4.566, *p* = 0.025) (Figure 4). A Bonferroni test indicated that the 10 min collagenase digestion resulted in a reduction in hydroxyproline crosslinking (*p* = 0.047). However, the 30 min collagenase digestions did not have a significant reduction in hydroxyproline compared to the 10 min collagenase digestions (*p* = 1.000) or CTRL group (*p* = 0.072).

## 4 DISCUSSION

Assessment of collagen in vivo remains a challenge, especially when cost, patient or experimental model considerations, and serial assessment are considered. Echocardiography provides an appealing diagnostic modality given its noninvasive and low‐cost implementation, along with its limited contraindication. Efforts are being made to use ultrasonic backscatter to understand collagen content in patients (Hoyt et al., 1984; O'Donnell et al., 1981; Rijsterborgh et al., 1996; Zaidman et al., 2008), but were limited to a specific myocardial position. Myofiber architecture and mesostructure of the heart are gaining interest, especially when applied to MRI (Wilson et al., 2022). Our previous studies have suggested that the myofiber architecture in the heart could be determined using ultrasonic backscatter (Milne et al., 2012, 2016, 2019), but it was unclear whether collagen or muscle was a primary determinant of this measure. The current study integrates collagenase treatment, a common method in preclinical research, with a novel application of ultrasound. In brief, we digested rat hearts with a collagenase solution and assessed whether changes in ultrasonic backscatter intensity and anisotropy are related to changes in collagen content and crosslinking.

### 4.1 Collagen and ultrasonic backscatter

In our study, collagenase, delivered via Langendorff perfusion, increased backscatter intensity throughout the tissue and reduced backscatter anisotropy within the myocardium in a nonuniform pattern. Prior to digestion, the most intense ultrasonic backscatter was visualized in areas of the myocardium where myofibers aligned perpendicular to the angle of insonification: backscatter intensity was comparatively reduced in areas of the myocardium with predominately parallel myofibers. This fiber‐alignment determinant of the anisotropy of ultrasonic backscatter has been previously validated (Milne et al., 2012, 2016, 2019). Throughout collagenase digestion, backscatter intensity increased disproportionally in areas where myofibers aligned parallel to the angle of insonification. Our integrated ultrasonic backscatter intensity data also suggests that collagen network orientation is an important consideration when assessing integrated backscatter in the myocardium.

Collagenase is typically used for tissue dissociation and cell isolation (Louch et al., 2011). While we used the same steps and solutions typically used in a myocyte isolation (Czeiszperger et al., 2020), we did not triturate or mechanically disrupt the hearts, which stayed intact but softened under collagenase treatment. Our choice of collagenase contains proteases and thus, myocytes themselves could be damaged. However, muscle birefringence imaging, which has previously been shown to be altered by cardiac thermal injury (Whittaker et al., 2000), was unchanged and striations remained evident (Figure 3). Therefore, we conclude that damage to the myocytes is unlikely to be the primary cause of the loss in ultrasonic anisotropy over the course of the treatment.

The collagen itself does not appear to washout from the myocardium, evidenced by the constant collagen‐to‐tissue ratio throughout collagenase digestion, but myocardial structure does appear to be disrupted. The increased angular deviation of the orientation distributions was consistent with disarray. A lack of coherent alignment has previously been associated with a lack of a collagen framework (Whittaker et al., 2003). However, lack of changes in myocyte birefringence and the qualitative appearance of a scattered, tinsel‐like appearance in imaging results leads us to propose that this change in backscatter is due to change in collagen organization and/or structure. The damage could have two components: damage to collagen struts, unraveling of collagen fibers, or a combination of these factors. Collagen struts provide lateral connection between neighboring myocytes (Weber et al., 1987, 1994). Independent of changes in collagen content, breakage of such struts will allow “slippage” of myocytes which, when combined with edema, will result in loss of the normal highly coherent myocyte alignment.

We further investigated whether the color (hue) of the polarized light would provide any additional information about changes to the collagen structure. We observed no statistical significance. In contrast, collagen fibers along the length of myocytes are typically formed by crosslinking of multiple fibrils. The reduced hydroxyproline observed in this study supports the reduction of crosslink quantity paired with collagenase digestion of the myocardium. While hydroxyproline bonds are present in all collagen fibrils, it has been shown that crosslinking will increase the output of this assay (Filova et al., 2020; Kato et al., 1995). While these data indicate that collagen crosslinking is generally reduced, collagen struts that are under tension in the intact heart may see the largest histological change. In contrast, the collagen fibrils along the length of the myocytes may not shift, unravel, or breakdown into thinner fibers. These data suggest that collagen crosslink disruption is the primary outcome of collagenase treatment.

The exact method by which collagenase digestion eliminates collagen crosslinks and degrades collagen structure was not evaluated by this study. However, prior reports suggest that collagen fibrils may become perforated, segmented, or disfigured as collagen fibrils become un‐crosslinked and the extracellular matrix loses integrity (MacKenna et al., 1994). The loss of integrity is evidenced in several of our histological slices (Figure 3). These data suggest that the ECM structure itself may be responsible for the attenuation of backscatter and the anisotropy. A static, patent matrix surrounding cells may perhaps act as a wave guide, transmitting instead of scattering the ultrasound. However, a more likely explanation is that the patent matrix lacks interfaces for ultrasound to backscatter. Disruption of ECM structure would create more interfaces for ultrasound scattering and explain the overall more intense backscatter and loss of anisotropy throughout the myocardium.

**FIGURE 5:**
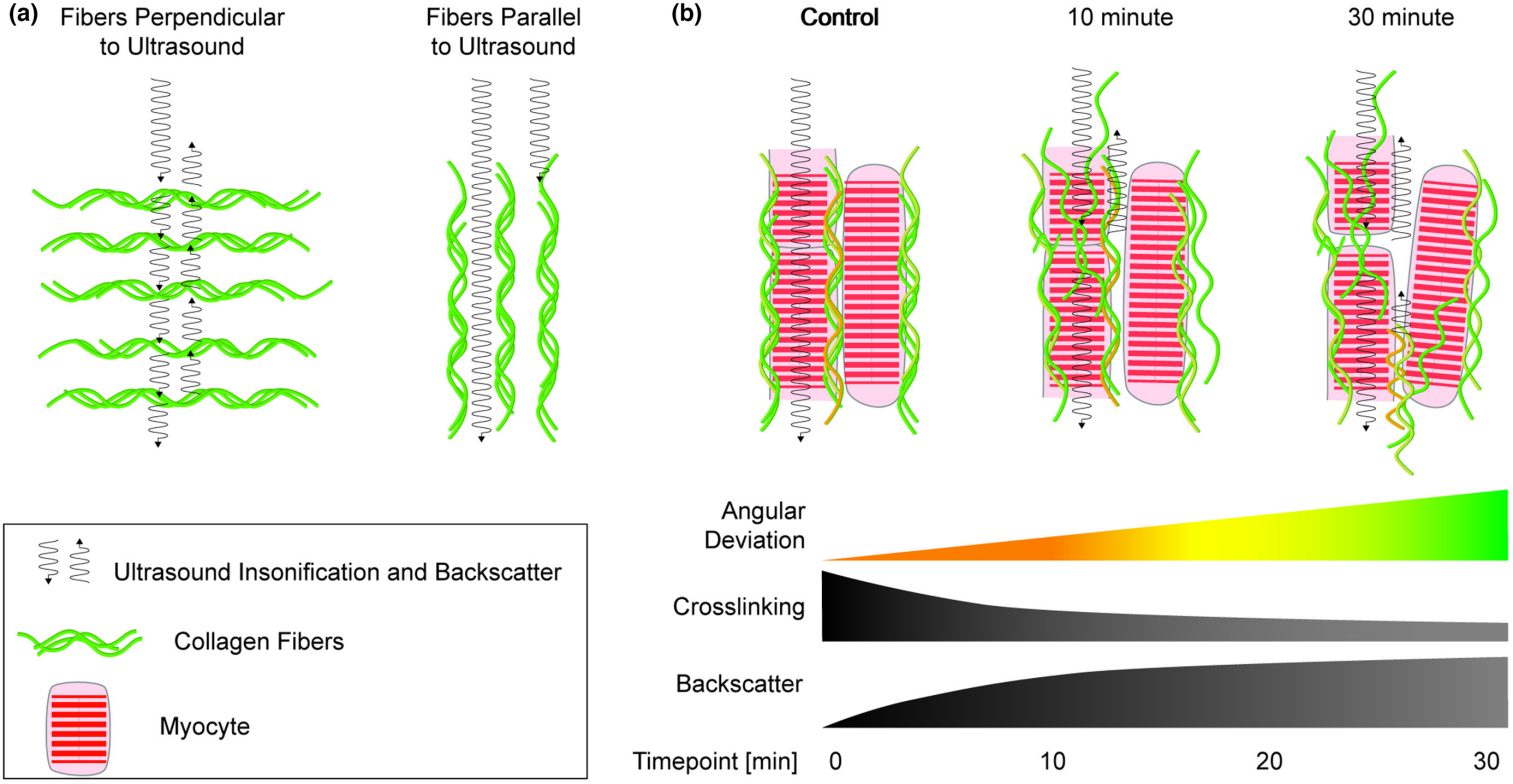
Effect of collagen orientation and crosslinking on ultrasonic backscatter. (a) Increased ultrasonic backscatter is generated by collagen oriented perpendicular to the angle of insonification. When ultrasound is insonified parallel to the collagen fibers, the reflection back to the ultrasound source (backscatter) is reduced. (b) In the myocardium as described with the data in this study, collagenase treatment corresponds with increased angular deviation of the collagen fibers, and possibly the myocytes, and a reduction in collagen crosslinking. This is related to an increase in ultrasonic backscatter, likely generated by the fibers that are no longer aligned with the angle of insonification. A reorientation of fibers toward perpendicular (compared to the angle of insonification) would result in increased ultrasonic backscatter.

Combined evaluation of our collagenase duration and timepoint interaction and our colorimetric hydroxyproline assay suggests a correlation between the timing of collagen degradation and changes in backscatter intensity and its anisotropy. The first 10 minutes of collagenase digestion created the greatest reduction in hydroxyproline content as well as the greatest changes in mean ultrasonic backscatter. These conclusions suggest that the interaction between collagen and ultrasound can be summarized as the following: ultrasound that interacts with collagen aligned perpendicular to the angle of insonification (the direction of the ultrasound wave), will generate substantial backscatter (Figure 5a). Ultrasound that interacts with collagen aligned parallel to the angle of insonification will not immediately reflect back to the transducer and will instead travel along the myofibers before the ultrasound encounters a new scatterer, such as perpendicularly aligned collagen. We cannot exclude from the current data any scattering away from the transducer, but the collagen could be acting like a wave guide allowing the ultrasound to travel away from the transducer. Further, when integrated into the tissue (Figure 5b), treatment with collagenase reduces collagen crosslinks and supports the unraveling of fibers. Collagen struts between fibers also begin to degrade. These changes are consistent with an increase in backscatter where collagen is aligned parallel to the angle of insonification, resulting in a decrease in the anisotropy index.

### 4.2 Anisotropy Index

Prior studies suggest that the absolute backscatter of a tissue correlates with total collagen content or deposition (Glueck et al., 1985; Hoyt et al., 1984, 1985; Kwan et al., 2022; Rijsterborgh et al., 1996), but such measures typically only quantify backscatter of the posterior wall, where myofibers lay perpendicular to the angle of insonification. The anisotropy of ultrasonic backscatter is a trademark of a healthy myocardium, striated muscles, and tendons (Aygen & Popp, 1987; Holland et al., 2005; Milne et al., 2012, 2016, 2019; Zaidman et al., 2008), but whether the anisotropy was generated by collagen or cellular materials was not previously known. The data in our study suggest that a more aligned and cross‐linked collagen network is related to an increase in anisotropy of the ultrasound signal and thus an increase in the Anisotropy Index developed in this study.

Our treatment did not show clear changes in the backscatter of the posterior (or anterior) left ventricular myocardium, but a change in backscatter was observed in the mid‐wall of the lateral and septal myocardium. While statistically significant, we propose a differential Anisotropy Index that leverages the high brightness myocardium perpendicular to the angle of insonification as a reference to assess for changes in collagen structure over time. Our ex vivo study allowed for a design that eliminates factors that may alter in vivo backscatter, such as muscular tissues, bone, and fat that might induce attenuation. The Index also enables the possibility of standardization per patient, rather than a population control, that is, changes in a specific subject's Anisotropy Index would indicate remodeling of the collagen network.

The Anisotropy Index leverages the fiber orientation in a heart and uses a single short‐axis image. In our presented data, all four calculated Anisotropy Indexes did exhibit differences in the statistical results, including difference over time. A single‐plane measurement does assume that the collagen remodeling is uniform, but this limitation could be addressed by adding more ultrasonic images with differing angles of insonification relative to a specific segment of the heart, or analysis of 3D images. The use of multiple images would allow for the Anisotropy Index to be used in other organ systems with linear alignment, such as vascular walls, skeletal muscles, and tendons (Baues et al., 2017; Zaidman et al., 2008). Nonetheless, the four calculated Anisotropy Index values and their average all follow the same relationship as the location did not reveal a statistical interaction with either the treatment or timepoint.

The experimental design and data presented here provide a proof‐of‐concept showing that anisotropy of ultrasonic backscatter in myocardial studies is likely related to cross‐linked collagen. While collagenase treatment is not viable as a treatment for fibrotic deposition in the myocardium, its use allowed us to alter the collagen structure of the myocardium. Future studies tracking changes in the Anisotropy Index during disease progression may provide additional insights and potentially provide a lower‐cost and noninvasive (biopsy‐free) method of tracking collagen remodeling in the heart.

## 5 CONCLUSION

The results from this study suggest that changes in collagen crosslinking may be detected and quantified serially using an Anisotropy Index, which quantifies ultrasonic backscatter intensity. The index utilizes two or more positions within myocardial walls to leverage fiber direction versus a traditional single‐site assessment, which amplifies the data we can collect from ultrasound. Ultrasound methods provide opportunities to visualize the structure and composition of many tissue types beyond the myocardium, as it is a noninvasive technology. Our proposed Anisotropy Index may provide a means to monitor and recognize cardiomyopathies related to collagen structure and myocardial fibrosis in vivo.

## FUNDING INFORMATION

The authors acknowledge Po‐Jen Chiang for support in histological and hydroxyproline assays. Supported by the National Institutes of Health (1R01HL151738, 1R01EB030058) to CSC, American Heart Association (18TPA34170169) to CSC, and the Patuxent Partnership to MLM.

## DISCLOSURES

An invention disclosure has been submitted by LAP, MLM, and CSC related to a method to quantify collagen structure via ultrasound derived Anisotropy Index.

## Data Availability

DOI for Supplemental Statistical Output: https://doi.org/10.6084/m9.figshare.21312117
